# Recent trends in 30-day mortality in patients with blunt splenic injury: A nationwide trauma database study in Japan

**DOI:** 10.1371/journal.pone.0184690

**Published:** 2017-09-14

**Authors:** Chie Tanaka, Takashi Tagami, Hisashi Matsumoto, Kiyoshi Matsuda, Shiei Kim, Yuta Moroe, Reo Fukuda, Kyoko Unemoto, Hiroyuki Yokota

**Affiliations:** 1 Department of Emergency and Critical Care Medicine, Nippon Medical School Tama Nagayama Hospital, Tokyo, Japan; 2 Department of Clinical Epidemiology and Health Economics, School of Public Health, The University of Tokyo, Tokyo, Japan; 3 Department of Emergency and Critical Care Medicine, Nippon Medical School Chiba Hokusoh Hospital, Chiba, Japan; 4 Department of Emergency and Critical Care Medicine, Nippon Medical School Musashikosugi Hospital, Kanagawa, Japan; 5 Department of Emergency and Critical Care Medicine, Nippon Medical School Hospital, Tokyo, Japan; University of Florida, UNITED STATES

## Abstract

**Background:**

Splenic injury frequently occurs after blunt abdominal trauma; however, limited epidemiological data regarding mortality are available. We aimed to investigate mortality rate trends after blunt splenic injury in Japan.

**Methods:**

We retrospectively identified 1,721 adults with blunt splenic injury (American Association for the Surgery of Trauma splenic injury scale grades III–V) from the 2004–2014 Japan Trauma Data Bank. We grouped the records of these patients into 3 time phases: phase I (2004–2008), phase II (2009–2012), and phase III (2013–2014). Over the 3 phases, we analysed 30-day mortality rates and investigated their association with the prevalence of certain initial interventions (Mantel-Haenszel trend test). We further performed multiple imputation and multivariable analyses for comparing the characteristics and outcomes of patients who underwent TAE or splenectomy/splenorrhaphy, adjusting for known potential confounders and for within-hospital clustering using generalised estimating equation.

**Results:**

Over time, there was a significant decrease in 30-day mortality after splenic injury (*p* < 0.01). Logistic regression analysis revealed that mortality significantly decreased over time (from phase I to phase II, odds ratio: 0.39, 95% confidence interval: 0.22–0.67; from phase I to phase III, odds ratio: 0.34, 95% confidence interval: 0.19–0.62) for the overall cohort. While the 30-day mortality for splenectomy/splenorrhaphy diminished significantly over time (*p* = 0.01), there were no significant differences regarding mortality for non-operative management, with or without transcatheter arterial embolisation (*p* = 0.43, *p* = 0.29, respectively).

**Conclusions:**

In Japan, in-hospital 30-day mortality rates decreased significantly after splenic injury between 2004 and 2014, even after adjustment for within-hospital clustering and other factors independently associated with mortality. Over time, mortality rates decreased significantly after splenectomy/splenorrhaphy, but not after non-operative management. This information is useful for clinicians when making decisions about treatments for patients with blunt splenic injury.

## Introduction

Splenic injury is one of the most frequent injuries after blunt abdominal trauma [1, 2]. There have been a few studies regarding mortality after splenic injury. Richardson [3] reported that, despite changes in the management of splenic injury, total mortality remained at 6–7%. Cirocchi et al. [4] reported that mortality after splenic injury was 14% in patients treated with non-operative management (NOM) and 17% in patients treated with splenectomy/splenorrhaphy. According to a large cohort study using a nationwide trauma database, the overall in-hospital mortality in the United States was 6.1% [5]. However, these previous studies did not investigate the epidemiologic trends in mortality for splenic injury patients.

The strategy for management of blunt splenic injury, which may have an impact on mortality, has changed in the last decade. Until the 1990s, splenectomy/splenorrhaphy was the standard treatment strategy for patients with blunt splenic injury. Subsequently, several studies reported that NOM with or without transcatheter arterial embolisation (TAE) has become more common in hemodynamically stable patients with blunt splenic injury, and mortality rates in such patients have decreased [6–9]. However, most of those reports came from single-centre or small-scale studies. Although several other reports have focused on the criteria for NOM indication and the cause of NOM failure in splenic injury patients, the trends in mortality were not fully investigated [3, 10–13]. Other factors that may influence mortality rates include the grade of splenic injury, as well as the incidence and nature of concomitant injuries. Nevertheless, there were few studies that investigated the relationship between mortality and the grade of splenic injury or associated injuries [6, 7].

We hypothesised that, over the last decade, mortality after blunt splenic injury has been decreasing. In the present study, we aimed to investigate the epidemiologic changes in the rate of in-hospital mortality among patients with blunt splenic injury in Japan, while adjusting for other factors related to mortality.

## Methods

The present study was approved by the ethics committee of the Nippon Medical School Tama Nagayama Hospital. The requirement for informed consent was waived because our analysis did not include personal identification information.

### Study design and data source

We conducted a retrospective cohort study using data from the Japan Trauma Data Bank (JTDB) [14–17]. The JTDB is a large national trauma database that is administered by the Japan Trauma Care and Research, and includes trauma cases classified as Abbreviated Injury Scale (AIS) [18] grade 3 or more, that were managed at 1 of 244 participating hospitals in Japan. The database contains data regarding the patients’ age, sex, vital signs on scene, vital signs at the emergency department, mechanism of injury, diagnosis, treatment, AIS scores, Injury Severity Score (ISS) [19], and survival [14–16].

### Definitions and variables

For spleen injuries, the AIS grades 3, 4, and 5 are equivalent, respectively, to grades III, IV, and V of the spleen injury scale proposed by the American Association for the Surgery of Trauma (AAST) [20]. A patient was defined to be under cardiac arrest on arrival if their respiratory and heart rates were zero. In addition to the baseline characteristics on scene and at the time of admission, several other variables were evaluated. We used the Japan Coma Scale (JCS) and the Glasgow Coma Scale (GCS) to evaluate the consciousness level [21, 22]. Further, we defined the initial management of splenic injury as the therapeutic interventions performed within 6 hours of arrival at the hospital. If patients had undergone both TAE and splenectomy/splenorrhaphy, we grouped them with the group of patients with splenectomy/splenorrhaphy.

The standard management strategy for blunt splenic injury has changed in the past decades, with the focus shifting from splenectomy/splenorrhaphy to NOM. There have been 3 major guidelines regarding the management of blunt splenic injury issued in the last decade. In 2003, the Eastern Association for the Surgery of Trauma (EAST) [23] published the first practice guidelines for the NOM of blunt injury to the liver and spleen. Next, the Western Trauma Association (WTA) [24] addressed the critical decisions about the management of adult blunt splenic injury. The WTA guidelines recommended that TAE of the splenic artery might serve as adjunctive therapy in the NOM of splenic injury patients. The EAST management guidelines were revised in 2012 as follows [25]: routine splenectomy/splenorrhaphy was no longer indicated in hemodynamically stable patients, and angiography would be considered for patients with severe splenic injury and presence of a contrast blush on CT scan. We considered that the treatment of splenic injury might have changed as the guidelines changed, estimating a delay of at least 1 year between the issue of the guidelines and change in clinical practice. Therefore, we stratified patients according to the following 3 phases. The first was phase I (2004–2008), starting 1 year after the issue of the first EAST NOM guidelines in 2003 and before the adoption of the WTA critical decisions in 2008. The second was phase II (2009–2012), starting 1 year after publication of the WTA critical decisions in 2008 but before adoption of the revision of the EAST guidelines in 2012. The third was phase III (2013–2014), starting 1 year after the revision of the EAST guidelines in 2012.

### Patient selection

The present study included blunt splenic injury patients registered in the JTDB between 2004 and 2014, and whose splenic injury was classified with an AIS code of 3 or more. We selected patients aged 15 years and above. We excluded patients who were under cardiac arrest on arrival.

After stratifying the patients according to phases I, II, and III, we compared the data regarding patients with isolated splenic injury against data regarding multiple injury patients who also had splenic injury, in order to assess the impact of concomitant injuries on the outcomes of the treatment. The other terms used to designate the region of the trauma were head, chest, abdomen, spine, neck, face, and periphery (peripheral injury), according to the AIS classification [18]. Finally, we evaluated the association between mortality and the initial management of splenic injury (NOM without TAE, NOM with TAE, or splenectomy/splenorrhaphy).

### Outcome measures

The primary outcome measure was 30-day all-cause in-hospital mortality.

### Statistical analysis

We compared the patients’ background characteristics, treatment, and mortality over time (i.e., phases I, II, and III) using the Mantel-Haenszel trend test. One-way analysis of variance was used for continuous variables as appropriate. We further performed multiple imputation and multivariable analyses for comparing the characteristics and outcomes of patients who underwent TAE or splenectomy/splenorrhaphy, adjusting for known potential confounders and for within-hospital clustering. Only with complete and available case analyses provide inefficient, though valid, results when missing data are missing completely at random, but biased results when missing data are missing not at random (MNAR) or missing at random (MAR) [26–28]. A multiple imputation approach leads to unbiased results with correct standard errors, in situations where missing data are either MNAR or MAR [26–28]. Therefore, we performed multiple imputation and handled with missing data appropriately for the multivariate analysis. First, we performed multiple imputation [26, 27] whereby each missing value was replaced with a set of 5 substitute plausible values, in order to reduce bias caused by incomplete data. A multivariable regression model was constructed for each imputed data set, and the results of the 5 imputed data sets were combined into a single model, from which the statistical inference was taken. Second, we analysed the temporal changes in the primary outcome using a multiple logistic regression model adjusted for the within-hospital clustering effect using generalised estimating equation. We also adjusted for factors independently associated with mortality as suggested by previous studies (i.e. age, gender, splenic injury grade, Injury Severity Score, time from emergency call to hospital arrival, time from hospital arrival to intervention, conscious level on admission, systolic blood pressure, type of injury, and intervention type [7, 29–31]). We assumed that the time from arrival to intervention was one of the most important factors associated with mortality. Since patients treated with NOM without TAE cannot be associated with the variable of “time from arrival to intervention”, we did not include patients with NOM without TAE in this logistic regression analysis. The statistical significance threshold was set at *p* < 0.05. All analyses were carried out using IBM SPSS version 23 (IBM Corp., Armork, NY, USA).

## Results

**Fig 1:**
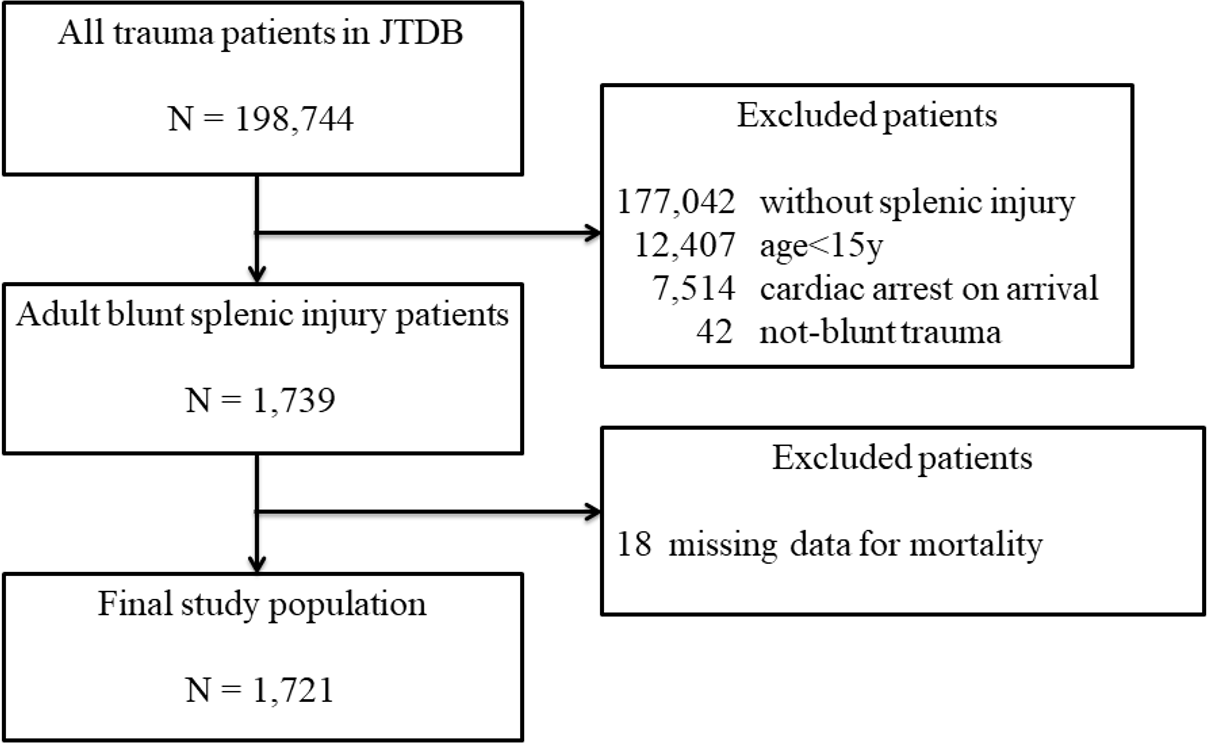
Patient selection.

During study phases I, II, and III, respectively, there were: 528, 1019, and 459 patients with AAST splenic injury grade 3 or more; 23, 42, and 13 patients with cardiopulmonary arrest on arrival; and 89, 106, and 139 participant hospitals. A total of 1,721 patients were selected (Fig 1), with 444 patients in phase I, 615 in phase II, and 659 in phase III. The basic characteristics, severity of the injury, pre-hospital information, and in-hospital information are summarised in Table 1. The mean ISS values were similar among the 3 phases (*p* = 0.33); besides, there were no significant differences in the proportions of patients with a given AAST splenic injury grade (*p* = 0.16). The proportion of patient who needed transfusion within 24 hours after injury significantly decreased over time (*p* = 0.01). Finally, there were no statistically significant differences regarding pre-hospital vital signs.

**Table 1:** Demographic and clinical characteristics of patients with blunt splenic injury, stratified by time periods.

**Variables**		**Phase I** **(n = 444)**		**Phase II** **(n = 615)**		**Phase III** **(n = 659)**		***p*-value**
Age, years		35.0	(22.0–56.0)	37.5	(24.0–62.0)	39.0	(23.0–61.0)	0.009
Male sex		332/444	(74.8)	450/615	(73.2)	482/659	(73.1)	0.57
Injury severity score		25.0	(17.0–36.0)	27.0	(17.0–37.5)	25.0	(16.0–34.0)	0.33
AAST Splenic Injury Scale grade								0.16
	3	264/444	(59.5)	404/615	(65.7)	418/659	(63.4)	
	4	138/444	(31.1)	164/615	(26.7)	193/659	(29.3)	
	5	42/444	(9.5)	47/615	(7.6)	48/659	(7.3)	
Isolated splenic injury		129/444	(29.1)	183/614	(29.8)	213/659	(32.3)	0.23
Splenic injury with head injury		93/444	(20.9)	138/614	(22.5)	118/659	(17.9)	0.16
Splenic injury with chest injury		245/444	(55.2)	348/615	(56.6)	371/659	(56.3)	0.74
Splenic injury with abdominal injury		94/444	(21.2)	100/615	(16.3)	108/659	(16.4)	0.06
Splenic injury with spine injury		21/444	(4.7)	30/615	(4.9)	31/659	(4.7)	0.97
Splenic injury with neck and face injury		2/444	(0.5)	5/615	(0.8)	4/659	(0.6)	0.81
Splenic injury with peripheral injury		108/444	(24.3)	137/615	(22.3)	126/659	(19.1)	0.04
Pre-hospital Japan Coma Scale score								0.81
	0	161/355	(45.4)	194/473	(41.0)	212/502	(42.2)	
	1	94/355	(26.5)	155/473	(32.8)	150/502	(29.9)	
	2	36/355	(10.1)	48/473	(10.1)	55/502	(11.0)	
	3	64/355	(18.0)	76/473	(16.1)	85/502	(16.9)	
Pre-hospital systolic blood pressure		110	(94–133)	110	(92–130)	112	(94–131)	0.22
Pre-hospital pulse rate		90	(73–104)	90	(74–107)	90	(77–105)	0.39
Pre-hospital respiratory rate		24	(18–28)	24	(18–30)	24	(20–30)	0.92
Time from emergency call to hospital arrival		35	(27–47)	38	(30–55)	37	(29–50)	0.03
In-hospital Japan Coma Scale								0.21
	0	172/366	(47.0)	228/499	(45.7)	244/503	(48.5)	
	1	72/366	(19.7)	127/499	(25.5)	113/503	(22.5)	
	2	54/366	(14.8)	65/499	(13.0)	81/503	(16.1)	
	3	64/366	(17.5)	79/499	(15.8)	65/503	(12.9)	
In-hospital Glasgow Coma Scale								0.76
	3–8	71/422	(16.8)	94/590	(15.9)	81/634	(12.8)	
	9–14	124/422	(29.4)	198/590	(33.6)	211/634	(33.3)	
	15	227/422	(53.8)	298/590	(50.5)	342/634	(53.9)	
In-hospital systolic blood pressure		111	(90–131)	110	(87–129)	113	(96–133)	0.006
In-hospital heart rate		94	(78–115)	92	(75–111)	90	(76–108)	0.005
In-hospital respiratory rate		24	(20–30)	24	(20–30)	22	(19–28)	0.001
In-hospital body temperature		36.4	(35.8–36.8)	36.2	(35.5–36.7)	36.3	(35.8–36.8)	0.13
Computed tomography during ER evaluation		275/444	(61.9)	381/615	(62.0)	423/659	(64.2)	0.41
Time from arrival to splenectomy/splenorrhaphy		104	(70–163)	123	(84–172)	110	(76–157)	0.92
Time form arrival to TAE		110	(79–162)	111	(65–165)	104	(68–147)	0.42
Transfusion within 24 hours		191/444	(43.0)	270/615	(43.9)	239/659	(36.3)	0.01

Analysis based on records from the Japan Trauma Data Bank: phase I (2004–2008), phase II (2009–2012), and phase III (2013–2014). Data given as number of positive observations/total number of observations (percentage) or as median (interquartile range). For each variable, the number of missing observations can be obtained as the difference between the total number of patients in each phase and the total number of observations.

AAST, American Association for the Surgery of Trauma; ER, emergency room; TAE, transcatheter arterial embolization

**Fig 2:**
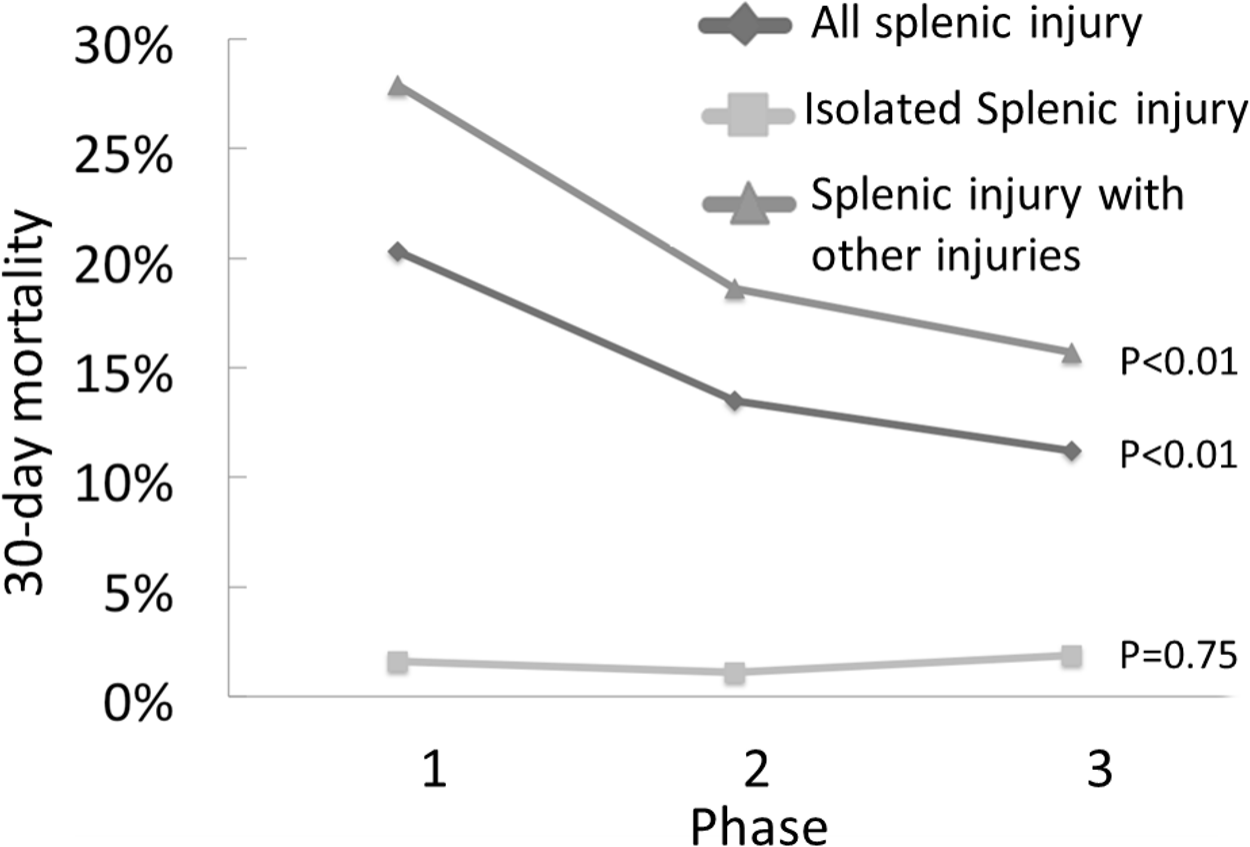
Comparison of 30-day mortality rates in patients with splenic injury, with or without associated injuries.

Fig 2 shows significant decreasing trends in 30-day mortality for all splenic injury patients and for splenic injury patients with multiple trauma across the 3 phases (*p* < 0.01 and *p* < 0.01, respectively). The 30-day mortalities in patients with isolated splenic injury were 2.5%, 0.6%, 1.8% for phase I, II, and III, respectively, and there was no significant difference among the phases *(p* = 0.75).

Table 2 shows the prevalence of the 3 forms of management for splenic injury in each phase. The management trends for isolated splenic injury were similar to those for the management of multiple injuries. The proportion of patients undergoing NOM with TAE significantly increased, and the proportion of patients undergoing splenectomy/splenorrhaphy significantly decreased over time (*p* = 0.01). The numbers of patients who underwent both TAE and an operative procedure for splenic injury were 4 (2.8%) in phase I, 9 (6.0%) in phase II, and 12 (9.1%) in phase III.

**Table 2:** Choice of initial management strategy for splenic injury, stratified by time periods.

	**Groups**	**Phase I**		**Phase II**		**Phase III**		***p*-value**
**Total patients**		(n = 444)		(n = 615)		(n = 659)		0.01
	**NOM without TAE**	230	(51.8)	348	(56.6)	372	(56.4)	
	**NOM with TAE**	71	(16.0)	116	(18.9)	155	(23.5)	
	**Splenectomy/Splenorrhaphy**	143	(32.2)	151	(24.6)	132	(20.0)	
**Isolated splenic injury**		(n = 129)		(n = 183)		(n = 213)		0.71
	**NOM without TAE**	86	(66.7)	125	(68.3)	137	(64.3)	
	**NOM with TAE**	19	(14.7)	30	(16.4)	58	(27.2)	
	**Splenectomy/Splenorrhaphy**	24	(18.6)	28	(15.3)	18	(8.5)	
**Splenic injury with other injuries**		(n = 315)		(n = 431)		(n = 446)		0.01
	**NOM without TAE**	144	(45.7)	223	(51.7)	235	(52.7)	
	**NOM with TAE**	52	(16.5)	86	(20.0)	97	(21.7)	
	**Splenectomy/Splenorrhaphy**	119	(37.8)	122	(28.3)	114	(25.6)	

Analysis based on records from the Japan Trauma Data Bank: phase I (2004–2008), phase II (2009–2012), and phase III (2013–2014).

Data given as total number (percentage).

NOM, non-operative management; TAE, transcatheter arterial embolization

Table 3 shows the 30-day mortality of the 3 forms of management for splenic injury in each phase. There was a significant trend towards decreasing 30-day mortality rates over the three phases for total splenic injury patients who underwent splenectomy/splenorrhaphy (*p* <0.01). On the other hand, there were no significant differences among the phases regarding 30-day mortality of patients managed by NOM with or without TAE.

**Table 3:** 30-day mortality of initial management strategy for splenic injury, stratified by time periods.

	**Groups**	**Phase I**		**Phase II**		**Phase III**		***p*-value**
**Total patients**		(n = 444)		(n = 615)		(n = 659)		
	**NOM without TAE**	28	(12.2)	44	(12.6)	36	(9.7)	0.29
	**NOM with TAE**	5	(7.0)	11	(9.5)	8	(5.2)	0.43
	**Splenectomy/Splenorrhaphy**	57	(39.9)	28	(25.0)	30	(23.6)	<0.01
**Isolated splenic injury**		(n = 129)		(n = 183)		(n = 213)		
	**NOM without TAE**	0	(0)	1	(0.8)	1	(0.7)	0.53
	**NOM with TAE**	0	(0)	0	(0)	0	(0)	0
	**Splenectomy/Splenorrhaphy**	2	(8.3)	1	(3.6)	3	(16.7)	0.40
**Splenic injury with other injuries**		(n = 315)		(n = 431)		(n = 446)		
	**NOM without TAE**	28	(19.4)	43	(19.3)	35	(14.9)	0.22
	**NOM with TAE**	5	(9.6)	11	(12.8)	8	(8.2)	0.66
	**Splenectomy/Splenorrhaphy**	55	(46.2)	26	(21.3)	27	(23.7)	<0.01

Analysis based on records from the Japan Trauma Data Bank: phase I (2004–2008), phase II (2009–2012), and phase III (2013–2014).

Data given as total number (percentage).

NOM, non-operative management; TAE, transcatheter arterial embolization

Logistic regression analysis adjusted for factors independently associated with mortality revealed that 30-day mortality significantly decreased over time (from phase I to phase II, odds ratio: 0.39, 95% confidence interval: 0.22–0.67; from phase I to phase III, odds ratio: 0.34, 95% confidence interval: 0.19–0.62) in patients treated either with TAE or with splenectomy/splenorrhaphy (Table 4).

**Table 4:** Multiple logistic regression analysis for risk of 30-day mortality among patients treated with transcatheter arterial embolization (TAE) or splenectomy/splenorrhaphy.

	**Original data set**				**After multiple imputation**		
	Odds ratio	95%CI		*p*-value	Odds ratio	95%CI	*p*-value
**Phase III (2013–2014)**	0.31	0.17–0.57		<0.001	0.34	0.19–0.62	<0.001
**Phase II (2009–2012)**	0.37	0.21–0.66		0.001	0.39	0.22–0.67	0.001
**Phase I (2004–2008)(reference)**	1				1		
**Male sex**	1.26	0.70–2.29		0.44	1.40	0.79–2.48	0.25
**Female sex (reference)**	1				1		
**Spleen injury grade 5**	1.45	0.67–3.12		0.35	1.63	0.73–3.65	0.23
**Spleen injury grade 4**	0.71	0.43–1.17		0.18	0.81	0.51–1.29	0.38
**Spleen injury grade 3 (reference)**	1				1		
**GCS on arrival 3–8**	8.98	4.52–17.84		<0.001	9.12	4.77–17.45	<0.001
**GCS on arrival 9–14**	2.73	1.59–4.70		<0.001	2.58	1.54–4.94	<0.001
**GCS on arrival 15 (reference)**	1				1		
**Age**	1.02	1.01–1.04		<0.001	1.03	1.01–1.04	<0.001
**Injury Severity Score**	1.03	1.01–1.06		0.002	1.04	1.02–1.06	<0.001
**Time from emergency call to hospital arrival**	0.99	0.97–0.99		0.005	0.99	0.97–0.99	<0.001
**Time from hospital arrival to intervention**	0.98	0.97–0.99		<0.001	0.98	0.99–1.00	0.003
**SBP on arrival**	0.99	0.98–1.00		0.011	0.99	0.98–1.00	0.003
**Injury type (Multiple)**	1.68	0.59–4.81		0.34	1.66	0.62–4.49	0.31
**Injury type (Isolated) (reference)**	1				1		
**Intervention type (Splenectomy/Splenorrhaphy)**	3.37	1.74–6.53		<0.001	2.84	1.56–5.17	0.001
**Intervention type (TAE) (reference)**	1				1		

Analysis based on records from the Japan Trauma Data Bank: phase I (2004–2008), phase II (2009–2012), and phase III (2013–2014).

GCS, Glasgow Coma Scale; SBP, Systolic Blood Pressure; TAE, Transcatheter Arterial Embolization. CI, confidence interval

## Discussion

The present study examined the records from a Japanese nationwide trauma registry to investigate the current trends in 30-day mortality of patients with blunt splenic injury. The results demonstrated that, in Japan, the all-cause 30-day in-hospital mortality rate decreased over a 10-year period (2004–2014) decreased significantly in patients with blunt splenic injury, even after adjustment for within-hospital clustering and other factors independently associated with mortality (e.g., splenic injury grade and ISS).

The strength of our study lies in its design, as it was based on records that contain pre-hospital information, severity of trauma injury, and in-hospital information from over 10 years of nationwide, multicentre experience (244 hospitals). A previous large-cohort study using a nationwide trauma database in the United States reported only overall mortality, without adjustment for other factors related to mortality [5]. Other previous multi-institutional retrospective studies regarding blunt splenic injury have focused mainly on the rates of NOM failure [11, 32–34]. Therefore, our study is the first nationwide cohort study that demonstrated the epidemiologic time-related trends in mortality after blunt splenic injury.

Compared to previous reports, our data suggested higher mortality rates after splenic injury[3–5, 35], which is likely related to the fact that our study population included patients with splenic injury grade 3 or more, indicating higher severity and ISS grade. Our data showed a significant increasing trend in the proportion of patients treated with NOM (with or without TAE) between 2004 and 2014, with a decreasing trend in the proportion of patients treated with splenectomy/splenorrhaphy. The present study also determined the trend in 30-day mortality associated with each management strategy. The mortality of patients treated with splenectomy/splenorrhaphy tended to decrease, while the mortality of patients treated with NOM (with or without TAE) did not change across the phases. Nevertheless, a significant decrease in 30-day mortality was found for this period of time in the patients with splenic injury included in the present study. This decrease was significant even after adjusting for within-hospital clustering and other factors independently associated with mortality.

The Ministry of Internal Affairs and Communications on Japan reported that, in general, the time from emergency call to hospital arrival increased by about 10 minutes over the 10-year period evaluated in our study. Indeed, as shown previously, the time from emergency call to hospital arrival increased significantly for cardiac arrest cases [36, 37]. Our results of the logistic regression analysis showed that the time from emergency call to hospital arrival was one of the independent factors associated with a poor outcome. Importantly, our results suggest that the outcomes of patients with blunt splenic injury improved despite the increase in the time from emergency call to hospital arrival.

Hamlat and colleagues [5] previously reported that inclusive trauma systems appeared to improve the outcomes of patients with splenic injury in the United States. We speculated that the decrease in 30-day mortality noted between 2004 and 2014 in the current study may, at least in part, be related to the progress of emergency medical services in Japan [38]. First, there was an improvement in the system of pre-hospital trauma-care education. The Japan Prehospital Trauma Evaluation and Care [39] (JPTEC) course started in 2003 and aimed to train rapid on-site observation, urgent treatment, and timely transportation of trauma patients. A decade later, the number of certified JPTEC providers in Japan had increased to 37,392 [40]. Second, there was an improvement in education regarding initial trauma management. The Japan Advanced Trauma Evaluation and Care [38] (JATEC) education program started in 2002 with 171 doctors, a number that had increased to 8,643 by 2012 [41]. The purpose of JATEC was to educate doctors regarding the emergency room management of patients with severe trauma, including splenic injury. Based on our results and previous observations [38], it is likely that such trauma training programs account for part of the decrease in mortality rates observed between 2004 and 2014 in splenic injury patients.

In addition to the spread of education about prehospital care and initial trauma management, we thought that the decrease of mortality was partially due to the progress in trauma management itself. For example, the guidelines about bleeding and coagulopathy following major trauma were published in 2007 and updated in 2010, 2013, and 2016 [42]. In addition, other practice management guidelines were published by EAST and there have been many papers about resuscitation for trauma patients [43–45]. These might contribute to improving resuscitation for severe trauma patients. From our study, the proportion of transfusion within 24 hours after injury tended to dwindle over the 10-year period. Thus, we speculated that progress in initial trauma management, including treatment for bleeding and coagulopathy, may have contributed to the decrease of in mortality after splenic injury.

We also considered that the specific guidelines in place at the time of admission might have an effect on the choice of treatment strategy for splenic injury. The guidelines suggested that the patient’s physical condition and the results of CT scans were important factors in the decision regarding the treatment of splenic injury; for example, recent guidelines recommend NOM for hemodynamically stable patients [23–25]. In our study, patients were stratified based on the time period during which specific guidelines were in effect. We found that the proportion of patients treated with NOM indeed increased over time, while mortality decreased in splenectomy/splenorrhaphy patients. From these results, we surmised that patient selection for a certain management strategy improved over the years, in accordance with the guidelines. However, there were no data that would allow us to evaluate the direct connection between the guidelines in effect at the time of admission and the choice of treatment in individual cases. Thus, we were not able to determine whether the guidelines had a direct effect on the choice and outcome of the treatment. Further studies are warranted in this direction.

There were several limitations to the current study. First, although the present study was a nationwide database survey, sampling of the 244 participating hospitals was not randomised or population-based. Besides, the number of patients per year increased in each phase because the number of hospitals participating in the database increased over time. The choice of management strategy is seemingly reflective of practice in specific trauma centres, and the increase in the number of participating hospitals over time might have had some effect on the results obtained in this study. However, we used the logistic regression model adjusted for the within-hospital clustering effect to account for this possibility. Second, there is a possibility that the initial rate of NOM failure (i.e., patients who were initially treated with NOM but converted to splenectomy/splenorrhaphy after 6 hours) status, re-interventions, or intervention-related complications have affected the mortality after splenic injury. However, the JTDB contains neither information about these factors, advancements in health care with resource utilisation, prehospital administered care on scene, nor the cause of death, so that we could not evaluate directly the effect of the management for splenic trauma on the outcome of our study. Third, we could not assess the quality of the intervention (e.g., skills of the surgeon performing splenectomy/splenorrhaphy, devices used during TAE, etc.), that of the perioperative management, or the cause of death, as such data are not available in the JTDB. Additionally, TAE might have involved not only angioembolisation of spleen bleeding, but also that of liver or kidney bleeding, and we did not account for the potential effect of this additional angioembolisation on mortality. Finally, we could not evaluate the direct effect of NOM on 30-day mortality. As suggested in the guidelines [23, 24], NOM is indicated only for hemodynamically stable patients. Hemodynamic states (i.e., vital signs) tend to change dramatically in the early stages after splenic injury; however, the JTDB only contained information regarding vital signs upon admission to the emergency room. The comparison of 30-day mortality between patients managed with NOM and those managed with splenectomy/splenorrhaphy would not be reliable in the context of the data included in our analysis. Further studies are required to evaluate the effect of NOM on mortality after splenic injury.

## Conclusions

The results of our nation-wide study suggest that, although the severity of injuries in Japan remained at the same level between 2004 and 2014, in-hospital 30-day mortality after blunt splenic injury decreased significantly, even after adjustment for within-hospital clustering and other factors independently associated with mortality. This information is useful for clinicians when making decisions about treatments for patients with blunt splenic injury. Nevertheless, as the present study did not elucidate the complex causes underlying the observed trends, further studies are required to confirm our results.
